# Cationic Cyclopentadienyliron Complex as a Novel and Successful Nucleating Agent on the Crystallization Behavior of the Biodegradable PHB Polymer

**DOI:** 10.3390/molecules23102703

**Published:** 2018-10-19

**Authors:** Safaa H. El-Taweel, Arwa O. Al-Ahmadi, Omaima Alhaddad, Rawda M. Okasha

**Affiliations:** 1 Department of Chemistry, Taibah University, 30002 Al-Madinah Al-Munawarah, Saudi Arabia; rooor.oth@hotmail.com (A.O.A.-A.); alhaddad105@yahoo.com (O.A.); 2 Chemistry Department, Faculty of Science, Cairo University, Orman-Giza, P.O. 12613, Egypt

**Keywords:** cationic organoiron complex, bacterial Poly(3-hydroxybutyrate) (PHB), nanocomposites, spherulitic morphology, crystal structure, isothermal and non-isothermal crystallization, thermal stability, FTIR analysis

## Abstract

Cationic cyclopentadienyliron (CpFe^+^) is one of the most fruitful organometallic moieties that has been utilized to mediate the facile synthesis of a massive number of macromolecules. However, the ability of this compound to function as a nucleating agent to improve other macromolecule properties has not been explored. This report scrutinizes the influence of the cationic complex as a novel nucleating agent on the spherulitic morphology, crystal structure, and isothermal and non-isothermal crystallization behavior of the Poly(3-hydroxybutyrate) (PHB) bacterial origin. The incorporation of the CpFe^+^ into the PHB materials caused a significant increase in its spherulitic numbers with a remarkable reduction in the spherulitic sizes. Unlike other nucleating agents, the SEM imageries exhibited a good dispersion without forming agglomerates of the CpFe^+^ moieties in the PHB matrix. Moreover, according to the FTIR analysis, the cationic organoiron complex has a strong interaction with the PHB polymeric chains via the coordination with its ester carbonyl. Yet, the XRD results revealed that this incorporation had no significant effect on the PHB crystalline structure. Though the CpFe^+^ had no effect on the polymer’s crystal structure, it accelerated outstandingly the melt crystallization of the PHB. Meanwhile, the crystallization half-times (t_0.5_) of the PHB decreased dramatically with the addition of the CpFe^+^. The isothermal and non-isothermal crystallization processes were successfully described using the Avrami model and a modified Avrami model, as well as a combination of the Avrami and Ozawa methods. Finally, the effective activation energy of the PHB/CpFe^+^ nanocomposites was much lower than those of their pure counterparts, which supported the heterogeneous nucleation mechanism with the organometallic moieties, indicating that the CpFe^+^ is a superior nucleating agent for this class of polymer.

## 1. Introduction

The development of biopolymers has attracted great interest over the years as a replacement for petroleum polymers. The key feature of this interest arises from the detrimental impact of traditional polymers on the world’s ecosystems. As a result, many researchers seek to exploit the synthesis of polymeric materials from renewable resources that will not persist in the environment after disposal [1,2,3,4]. Successful examples in this field include the synthesis of thermoplastic starch, poly(hydroxyalkanoic acid)s (PHAs), poly(lactic) acid, and their blends or copolymers with other biopolymers [5,6].

Among the poly(hydroxyalkanoic acid)s, polyester of 3-hydroxybutyrate (PHB) is an attractive example of a biodegradable and biocompatible polymer [7,8], which could be accessible via bacteria as intracellular carbon and energy storage compounds and accumulated as granules in the cytoplasm of cells [9,10]. This thermoplastic polymer has also been recognized as one of the most promising biopolymers in packaging and biomedical applications [11,12].

A general comparison of biodegradable polymers to traditional classes revealed certain drawbacks that pose challenges to their commercial applications. For example, in the case of PHB, its manufacturability has been limited due to its stiff and brittle character, narrow processing window, and high cost [13,14,15]. Another obstacle is the slow crystallization rate of PHB [14,15], when processed under conventional injection, molding, and/or extrusion methods, which have resulted in poor dimensional stability and low tackiness.

Several strategies have been reported to overcome some of the aforementioned disadvantages [16,17]. One of these strategies involves the insertion of nucleating agents into the PHB matrix in order to manipulate its crystallization rate, which makes it competitive with synthetic thermoplastic polymers. Various reports have revealed that the addition of nucleating agents to PHB has resulted in a significant improvement in thermal, mechanical, and other physical properties. Examples of these nucleating agents have included the following: NH_4_Cl [18], melamine [19], boron nitride [20,21], talc [22,23], cyanuric acid [24], cyclodextrin [25], lignin [26], and thermally reduced graphene (TRG) [27]. Recent examples have incorporated nanofillers, such as multi-walled carbon nanotubes [28,29], TiO_2_, and ZnO nanoparticles [30], graphite nanosheets [31], and WS_2_ inorganic nanotubes [32].

Despite the prevalence of previous studies that have explored a tremendous number of nucleating agents, this topic continues to be researched in order to overcome the drawbacks that have been associated with such nucleating agents [33,34,35,36,37,38,39,40,41,42,43]. For instance, some of the nucleating agents face an agglomeration issue inside the polymer matrix, which is attributed to the variance in surface energy [35]. This usually occurs with slow solvent evaporation or via the interface of the inorganic filler with the organic polymeric matrix. As a solution, researchers have made several attempts to modify the polymer matrix–filler interface through the incorporation of coupling agents, such as silane, onto the filler surface, which can enhance the interfacial bonding strength of the composites and improved its dispersity [34,44,45]. Meanwhile, examining new classes of nucleating agents remains one of the most successful strategies.

Over the past two decades, our work has been focused on utilizing the cationic cyclopentadienyl iron moieties in designing novel classes of monomeric and polymeric materials [46,47,48,49]. The intense electron withdrawing ability of the positively charged iron center facilitates nucleophilic substitution and addition reactions on the arene rings and allows for the production of these macromolecules under mild conditions. Furthermore, the iron complex demonstrates several advantages over other π-coordinated metallic moieties due to its chemical versatility, stability, low cost, and low toxicity [50,51,52,53].

This work presents the first example of exploring the effect of the CpFe^+^ complex as a novel nucleating agent on the isothermal and non-isothermal crystallization behavior of PHB using Differential Scanning Calorimetry (DSC) and polarized optical microscopy (POM). The crystallization kinetics and the activation energy have also been analyzed using several models, such as Avrami, Ozawa, a combination Avrami and Ozawa model, and the differential isoconversional method of Friedman. 

## 2. Results and Discussion

### 2.1. Polarized Optical Microscopy

**Figure 1:**
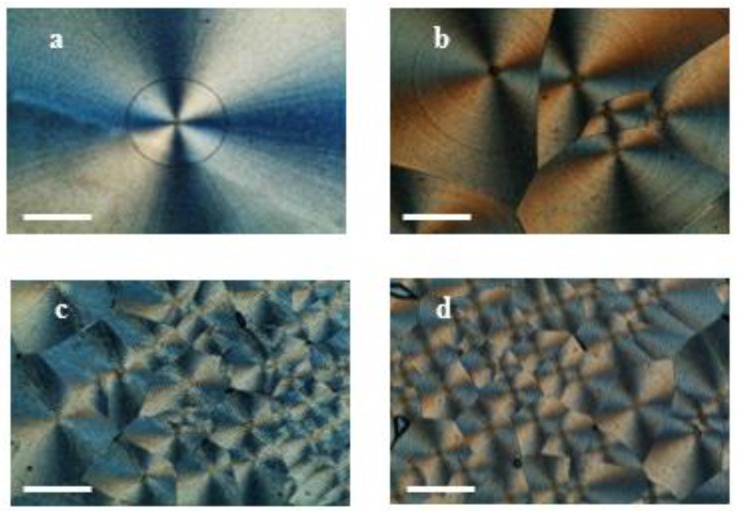
Polarized light optical micrographs of Poly(3-hydroxybutyrate) (PHB) spherulites of the pure PHB and PHB/CpFe^+^ nanocomposites with ratios of (**a**) 100:0, (**b**) 99.5:0.5, (**c**) 99:1, and (**d**) 97:3, respectively, after the isothermal crystallization at 100 °C scale bar 200 µm.

**Figure 2:**
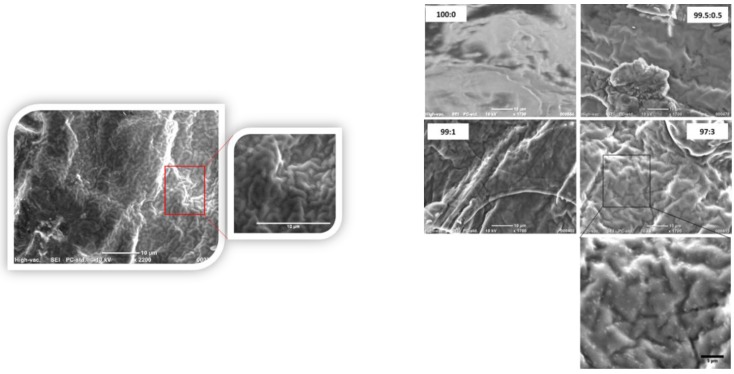
SEM micrographs of the pure PHB and PHB/ CpFe^+^ nanocomposites with 0.5%, 1%, and 3% of the CpFe^+^ moieties (right); zoomed-in image of the 97:3% of PHB/CpFe^+^ (left) that shows the helix structure with x 2200, scale bar 10 µm.

Figure 1 represents the polarized optical micrographs of the PHB and PHB/CpFe^+^ nanocomposites at the isothermal crystallization condition, 100 °C. The obtained results revealed that the size of the PHB’s spherulite was found to be as large as 1 mm, while the spherulite number was extremely small. This behavior could be attributed to the exceptional purity of the PHB that was prepared via bacterial origin [14]. It is also illustrated in Figure 1 that the PHB and PHB/CpFe^+^ nanocomposites displayed a Maltese cross section with regular extinction rings. The presence of the cationic cyclopentadienyliron (CpFe^+^) moieties led to the production of smaller spherulites than that existing in the pure PHB, where the CpFe^+^ nanoparticles were trapped in the center of the spherulites in the form of brown colored dots. Also, the CpFe^+^ particles were well distributed in the PHB matrix, which is clearly seen in the SEM images in Figure 2. Moreover, increasing the amount of the CpFe^+^ correspondingly increased the number of small spherulites. These results categorized the CpFe^+^ as one of the most highly efficient nucleating agents of PHB.

### 2.2. Scanning Electron Microscope (SEM)

The morphologies of the PHB and PHB/CpFe^+^ nanocomposites were examined using scanning electron microscope (SEM). Figure 2 elucidates the smooth, fractured surface of the pure PHB. Upon the combination of the cationic iron moieties with the PHB, the images revealed a remarkable distribution of the CpFe^+^ species without forming agglomerates in the polymer matrix. This behavior remained incessant while increasing the nucleating agent ratio. Additionally, the incorporation of the iron moieties into the polymer matrix triggered the formation of the helical structures of the polymer chains during the crystallization process, which can be observed in some of the SEM images [54,55,56].

### 2.3. FTIR Analysis

**Figure 3:**
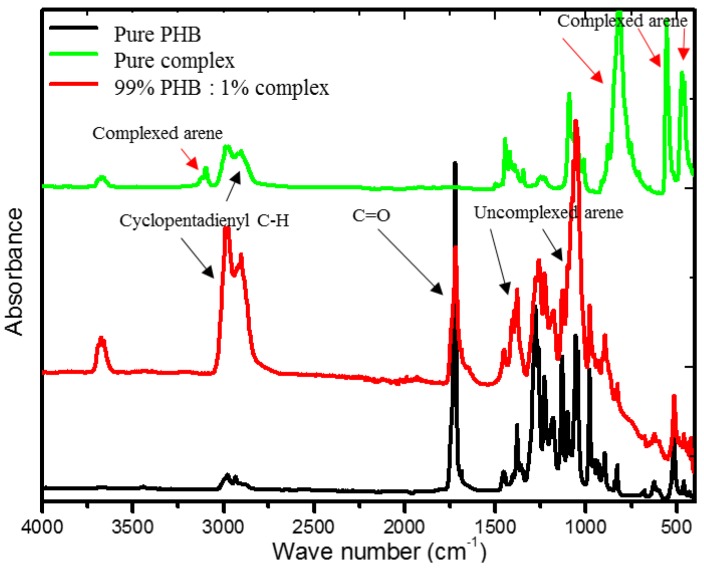
FTIR spectra of the CpFe^+^ complex, pure PHB, and PHB/ CpFe^+^ nanocomposites.

**Scheme 1:**
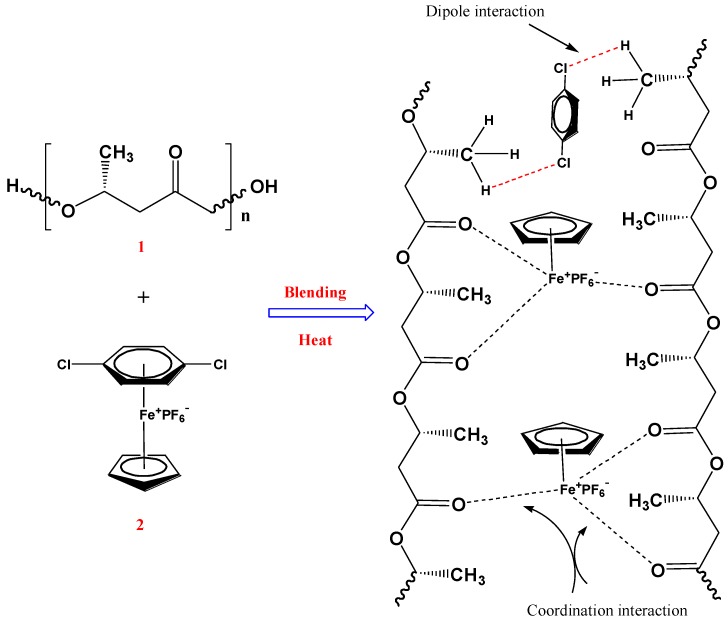
Proposed mechanism of the PHB/CpFe^+^ interaction.

The FTIR spectra of the CpFe^+^ moiety, pure PHB, and PHB/CpFe^+^ nanocomposites with a 99:1%, respectively, are illustrated in Figure 3. It is well known that the amorphous melt of the pure PHB can be crystalized during the cooling process, forming an antiparallel helical structure due to the formation of hydrogen bonds within the polymer matrix [55]. Meanwhile, preparing the blend sheets required the cast film samples to be molten between a hot melt press at 200 °C for 2 min. This process allowed for the decomplexation of the arene ring of the cationic iron moieties [47,48,49], which increased the possibility of forming a dipole interaction within the helical structure through the chlorine terminal atoms of the arene rings, as seen in Scheme 1. The decoordination of the arene ring could be confirmed via the FTIR spectra. For instance, the pure cationic cylopentadienyliron complex exhibited an out of plan bending peak, corresponding to the complexed arene C–H at 816 cm^−1^, while their stretching bands were observed at 469 and 553 cm^−1^. These peaks were shifted to a higher frequency, 1055 and 1410 cm^−1^, after the decoordination process and the blending with the PHB polymer; in addition, the spectrum exhibited a disappearance of the complexed arene C–H band at 3097 cm^−1^. On the other hand, the cyclopentadienyliron moieties became more susceptible to the coordination with the ester carbonyl of the polymer, as seen in Scheme 1. This finding has been verified via the obtained spectra. As can be seen from Figure 3, the characteristic peak at 1719 cm^−1^ in the pure PHB spectrum was assigned to the ester carbonyl group within the polymer matrix. Upon blending the polymeric materials with the CpFe^+^ complex, a decrease in the carbonyl stretching frequency was observed while maintaining its value, which confirmed the formation of a chelating complexation to the cationic iron moieties. Furthermore, the frequencies of the complexed cyclopentadienyl C–H stretching band and the CH_3_ bands of the PHB chains, in the range of 2901–2987 cm^−1^, were increased after the blending process with the preservation of their values, which supported the complexation behavior of the iron centers towards the PHB matrix

### 2.4. X-ray Results

**Figure 4:**
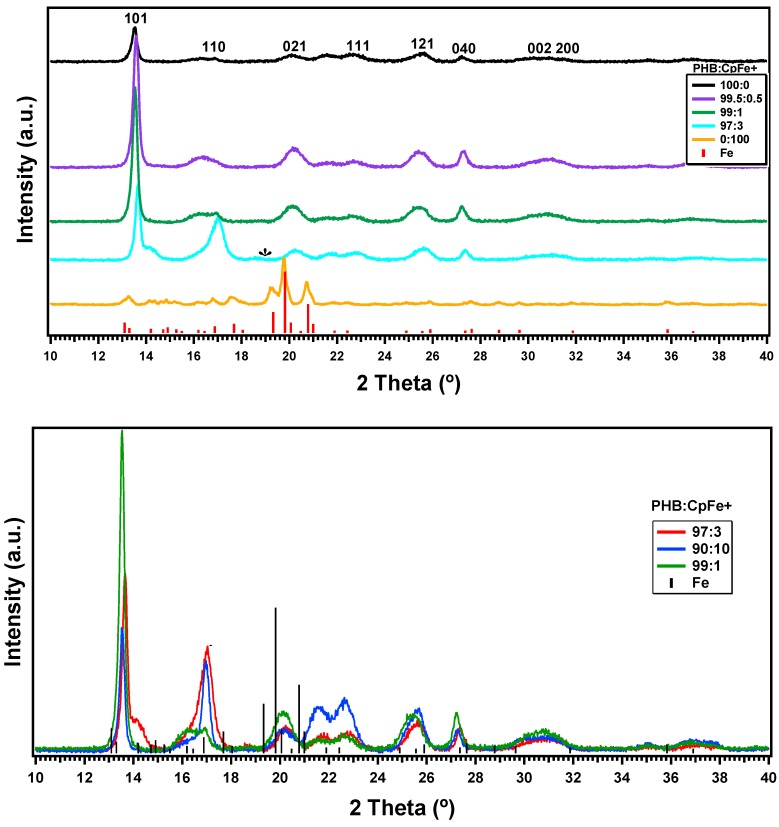
XRD pattern of pure PHB and PHB/ CpFe^+^ nanocomposites with ratios of 100:0, 99.5:0.5, 99:1, 97:3 respectively (**top**). XRD pattern of a higher ratio of CpFe^+^ 1%, 3%, and 10% (**bottom**).

Figure 4 shows the X-ray diffractograms of the pure PHB, CpFe^+^, and the PHB/CpFe^+^ nanocomposites. The characteristic Bragg reflection peaks of the CpFe^+^ complex can be observed at 2θ = 19.77, 20.08, and 21.48, which is indexed on the bases of the face centered cubic (FCC) structure of iron and found to be identical to those reported for the standard iron metal. The average crystal size of the CpFe^+^ was calculated, according to the Scherrer equation, and found to be around 42 nm. However, blending this organometallic complex with the PHB, followed by the thermal processing of the prepared sheets, enhanced the tendency of the iron center to coordinate with the ester carbonyl of the polymer as described previously and illustrated in Scheme 1. This coordination behavior of the CpFe^+^ was one of major factors that facilitated the excellent distribution of the metallic centers within the polymer matrix. Furthermore, the coordination process led to the disappearance of the diffraction peaks of the CpFe^+^ moiety at the (021) area. On the other hand, the metallic moieties exhibited a crystal matching with the polymeric materials that was evidenced by the perfect alignment of the diffraction peaks, which can be observed through the growth of its peaks at the (101) and (110) areas. It is also noticeable that the diffraction peaks of the PHB became sharper and narrower than the pure PHB ones with the increasing of the percentage of the iron complex, which is an indication of the increasing crystallinity of the modified polymer. Utilizing the Scherrer equation, the pure PHB exhibited a crystal size of 32.7 nm, while the PHB/CpFe^+^ nanocomposites displayed crystal sizes of 35.5, 36.8, and 37.2 nm for 0.5%, 1%, and 3% of the CpFe+ nanocomposites. In spite of the fact that the diffraction peaks of the modified polymeric materials retained their values, which suggested that the presence of the metallic moieties did not alter the structure of the polymer, increasing the ratio of the iron moieties reduced the intermolecular interactions between the C=O group and the CH_3_ group of the PHB due to the formation of a coordination interaction with the CpFe^+^ complex. This behavior resulted in the increase of the intensity and the narrowing of the (110) peak area with a value of 2θ (16.88°) (marked with asterisk). In order to verify the consistency of this performance, an extra ratio of the CpFe^+^ (10%) was blended with the PHB. Figure 4 (bottom) elucidates a comparison of the XRD patterns of the PHB blended with 1%, 3%, and 10% of the CpFe+ nanocomposites, which substantiates the previous findings.

### 2.5. Thermal Properties

**Figure 5:**
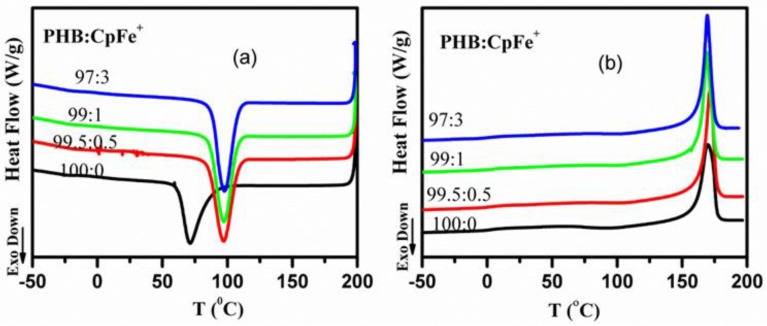
DSC curves of the pure PHB and PHB/CpFe^+^ nanocomposites; (**a**) cooling curves from the melt at a cooling rate 20 °C/min, (**b**) second heating curves with a heating rate 20 °C/min.

The Differential Scanning Calorimetry (DSC) cooling curves of the PHB and its nanocomposites are presented in Figure 5 and exhibited an exothermic peak corresponding to the PHB crystallization. It is well known that a higher value of the crystallization temperature, T_c_, is an indication of a faster crystallization rate. The values of the crystallization temperatures (T_c_) and the crystallization enthalpy (∆H_c_) are reported in Table 1.

The composition of the PHB nanocomposites manipulates the position and shape of the crystallization peak. The presence of the cationic iron (CpFe^+^) complexes in the PHB matrix resulted in a shift of the crystallization peaks to a higher temperature, Figure 5a, by 26 °C for all the PHB nanocomposites. The crystallization peaks of all the PHB nanocomposites maintained their values upon the addition of different ratios of CpFe^+^ complexes. These results could be attributed to the presence of the strong interaction between the organoiron centers (CpFe^+^) and the PHB matrix. As can be seen in Figure 5, the appearance of the crystallization peaks of the PHB nanocomposites became sharper and narrower compared with those of the pure PHB, which demonstrated that the CpFe^+^ acts as an efficient nucleating agent and suggested that the PHB crystallization occurs through a heterogeneous nucleating mechanism [18,19,20,21,22,23,24,25,26,27,28,29,30,31,32]. Therefore, the values of the ∆H_c_ for all the PHB nanocomposites were higher than those of the pure PHB. Figure 5b presents the DSC heating curves of the PHB and its nanocomposites, while the values of their glass transition temperature (T_g_), cold crystallization temperature (T_CC_), and melting temperature (T_m_) are listed in Table 1. The obtained results revealed that the values of the glass transition temperatures of the PHB and its PHB/CpFe^+^ nanocomposites were around 2 °C, which was independent of the PHB composition.

It has been established that the heat capacity of the glass transition temperature of amorphous polymers is much higher than those of crystalline ones. Thus, the values of the heat capacities for the PHB in its nanocomposites are lower than those of the pure PHB. Less intense broad exothermic cold crystallization peaks were detected for the nanocomposites during the second heating scan, as shown in Figure 5b and Table 1. These results revealed that the CpFe^+^ in the PHB nanocomposites prompted almost complete PHB crystallization during a cooling scan with 20 K/min. Meanwhile, above 150 °C, endothermic melting peaks were detected in the second heating scans, as shown in Figure 5b. The pure PHB and the PHB nanocomposites had similar values with respect to melting temperatures. These results indicated that the CpFe^+^ did not have a significant effect on the crystal structure of the PHB, which was in a good agreement with the XRD results, as shown in Figure 4. A similar trend was reported for the PHB with other nucleating agents [20,26,32,57]. It is worth mentioning that the width of the half height of the melting peak became narrower in comparison to the PHB. The degree of crystallinity was calculated from Equation (1) and reported in Table 1. In conclusion, the DSC results confirmed that the CpFe^+^ moieties had a significant increase in the degree of crystallinity, which suggests that the CpFe^+^ is an efficient nucleating agent for the PHB in PHB nanocomposites, as shown in Table 1.

(1) $$\;X_{c}=\frac{\Delta H_{m}-\Delta H_{cc}}{\Delta H_{m}^{{}^{\circ}}\times w}\;$$

### 2.6. Isothermal Crystallization Kinetics

**Figure 6:**
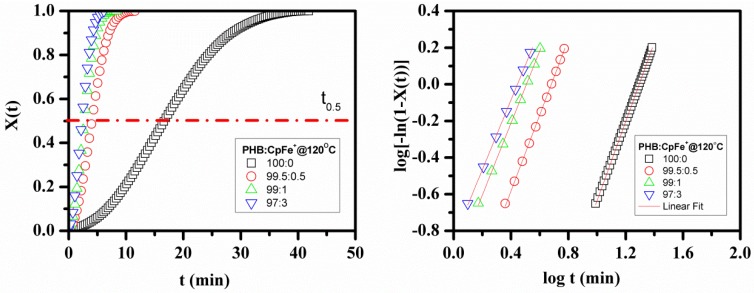
Relative crystallinity ( X(t)) (**right**) and its corresponding typical Avrami plot (**left**) in the isothermal crystallization process for the pure PHB and PHB/ CpFe^+^ nanocomposites.

The isothermal crystallization behavior of the pure PHB and its nanocomposites were investigated by rapidly cooling the melt (70 °C/min) to the selected crystallization temperature, ranging from 90° to 128 °C. The exothermic crystallization peaks were recorded as a function of the crystallization time. It is crucial to note that the crystallization peaks were broader at higher crystallization temperatures (Tc), indicating a lower crystallization rate. Figure 6 represents the relative crystallinity (X(t)) as a function of the crystallization time (t/min) at 120 °C, the selected crystallization temperature. X(t) was estimated by the analysis of the heat flow of the isothermal crystallization peaks with time, t, with the following Equation (2):

(2) $$\;X\left(t\right)=\frac{\int_{0}^{t}\frac{\partial H}{\partial t}\mathrm{dt}}{\int_{0}^{\infty }\frac{\partial H}{\partial t}\mathrm{dt}}\;$$

The numerator represents the integrated heat flow generated until time t, and the denominator is the total heat flow produced in the isothermal crystallization until t = ∞. It can be detected that in the X(t) range, between 0.2 and 0.8, the curves are straight. The isothermal crystallization kinetics of the PHB and its nanocomposites were analyzed based on the Avrami Equation, Equation (3):

(3) $$\;X\left(t\right)=1-\exp\left(-{\mathrm{kt}}^{n}\right)\;$$

The plot of (log(−ln(1−X(t))) as a function of log(t), derived from Equation (3), creates a linear line with the intercept and slope known as log k and n, respectively. n is the Avrami exponent, k is the overall crystallization kinetic rate constant, and t is the time of crystallization process. The values of k and n depend on the nucleation mechanism and the growth geometry. From Figure 6, it can be concluded that Avrami model, Equation (3), was applicable for describing the isothermal behavior of the PHB nanocomposites.

The crystallization parameters, *k*, *n*, and *t*_0.5,_ are all reported in Table 2. *t*_0.5_ is defined as the time at which the relative crystallinity is 50%. As seen in Table 2, *t*_0.5_ decreased with the decrease of the crystallization temperature and increased the percentage of the CpFe^+^ in the PHB/nanocomposites, which is a required behavior for processing the polymer materials. These results indicated that the rate of the PHB crystallization process is faster when the super cooling temperature is higher within the selected crystallization temperature range. At the given crystallization temperature, 120 °C, the *t*_0.5_ of the PHB crystallization process in the PHB nanocomposites was much lower than that of the pure analogues, while all the curves of the isothermal crystallization kinetics were almost parallel with each other, as shown in Figure 6. This revealed that the crystallization mechanism of the PHB in its nanocomposites was the same, where their *n* values were almost constant and approximately equal to 2. The value of *n* can be attributed to the presence of thermal crystallization during the crystallization process [58]. Another possible explanation reported in the literature [59] is that the PHB crystals can either develop sporadically as rods or instantaneously as disks. The rate constant, *k*, which is related to both the nucleation and the growth processes, increased with the decrease in the crystallization temperature and the increase of the ratio of the CpFe^+^ complex. For instance, at 120 °C, the crystallization rate of the PHB with the incorporation of 3 % of the CpFe^+^ moieties was found to be 87% faster than that of its pure analogue. These results verify the hypothesis that CpFe^+^ is a superior nucleating agent for this type of polymer.

### 2.7. Non-Isothermal Crystallization Kinetics

**Figure 7:**
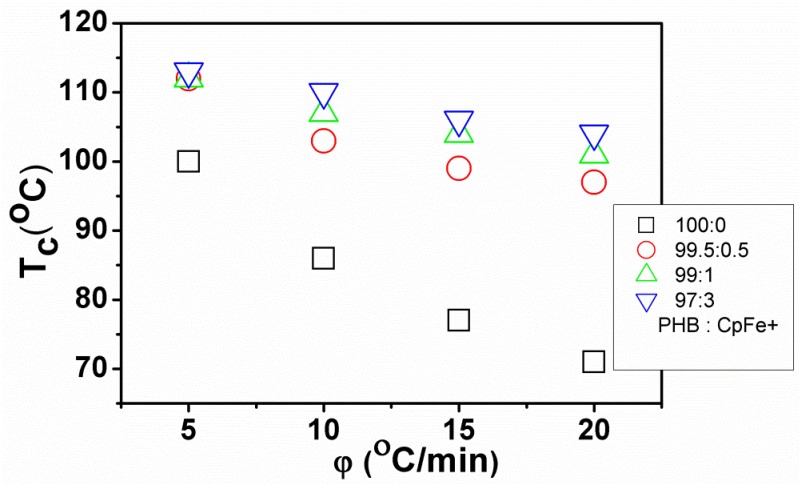
A maximum crystallization temperature as a function of a cooling rate for the non-isothermally crystallized pure PHB and PHB/ CpFe^+^ nanocomposites.

In the polymer processing techniques, the temperature changes continuously. Therefore, investigating the crystallization kinetics under non-isothermal conditions was essential. In general, the exothermic crystallization peak becomes broader and shifts to lower temperatures with the increasing of the cooling rate of all the PHB samples. The dependence of the crystallization temperature (Tc) on the cooling rate for the PHB/nanocomposites is shown in Figure 7.

**Figure 8:**
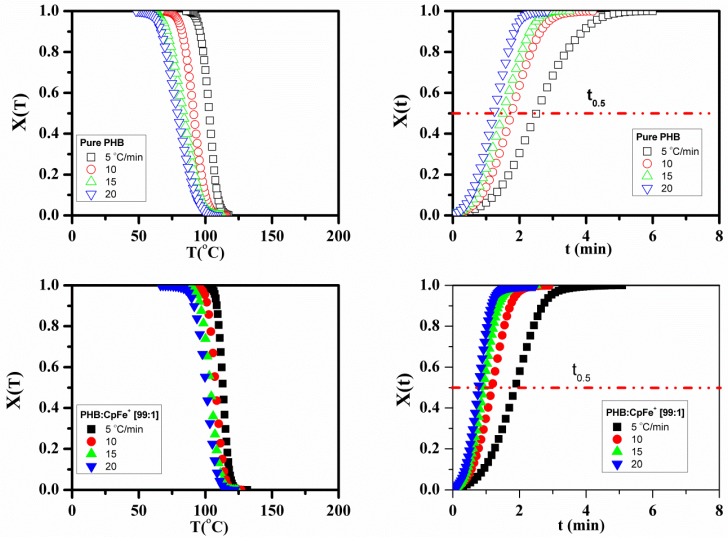
Relative crystallinity as a function of crystallization temperatures and time in the non-isothermal crystallization process for the pure PHB and PHB/CpFe^+^ nanocomposites at various cooling rates.

The attained data disclosed that the crystallization temperature of the PHB in its nanocomposites was higher than those of the pure counterparts for all the studied cooling rates. These results indicated that the enhancement of the crystallization temperature arises from the presence of the CpFe^+^ moieties. The relative crystallinity X(T) was evaluated from the DSC experimental data in Figure 8 by applying Equation (4), as follows:

(4) $$\;X\left(T\right)=\;\frac{\int_{T_{0}}^{T}\left(\frac{\partial H}{\partial t}\right)\partial t}{\int_{T_{0}}^{T_{\infty }}\left(\frac{\partial H}{\partial t}\right)\partial t}\;$$

The T_0_ and T_∞_ are the onset and the end crystallization temperature, respectively, and the ∂ H is the enthalpy of the process while the temperature is converted into the time scale, using Equation (5) as follows:

(5) $$\;t=\;\frac{\left|T-T_{0}\right|}{\varphi }\;$$

The T is the temperature at the crystallization time, and the φ is the cooling rate. The relative crystallinity (X(t)) versus the time curves is plotted in Figure 8 where all the curves exhibited the same sigmoidal shape and are shifted to a shorter time with increasing the cooling rates.

The Avrami model [60], the Ozawa model [61], and the combination of the Avrami and Ozawa models (the MO approach) are most frequently used to analyze the non-isothermal experimental crystallization data [62]. Regarding the cooling rate (φ), Jeziorny stated that the value of k*_t_* should be effectively corrected [63], and it can be calculated by Equation (6), as follows:

(6) $$\;\log k_{c}=\;\frac{\log\left(k_{t}\right)}{\varphi }\;$$

**Figure 9:**
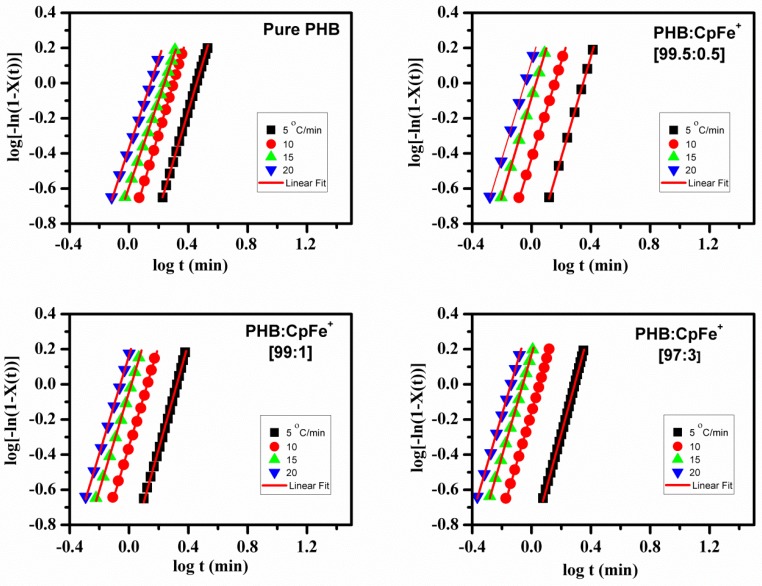
Typical Avrami plots of log(1−ln(1−X(t)) versus log(t) of the pure PHB and PHB/CpFe^+^ nanocomposites that non-isothermally crystallized at various cooling rates.

Figure 9 displays plots of log(−ln(1−X(t))) versus log(t). The non-isothermal parameters, crystallization rate constant ($k_{t}$), and Avrami exponent (n) were estimated by inserting the experimental data of the X(t) to be in the range of 0.2–0.8, using Equation (3), as listed in Table 3. The Avrami exponent values, n, for the non-isothermal crystallization process were found to be approximately 3, which was higher than those of the isothermal crystallization for both the pure PHB and PHB nanocomposites.

It is well known that the Avrami equation assumes that during the isothermal crystallization process, the nucleation rate remains constant. However, the nucleation rate is not constant when the PHB samples are crystallized under non-isothermal crystallization conditions [26]. Therefore, the n values for the non-isothermal crystallization kinetics are higher than those under the isothermal ones. This infers that much more complicated processes occur during the melt of non-isothermal crystallization [58]. Other possible explanations that appear in the literature are that n and k_t_ are two adjustable parameters [62,63,64]. Additionally, the k_t_ values, at any given composition, increase by increasing the cooling rate and, at any given cooling rate, increase with the increase in the content of the CpFe^+^. A similar trend was detected for an inverse half-life time of crystallization, as shown in Table 3.

Combining both the Avrami and Ozawa models was proposed by Liu [62] at a given value of relative crystallinity X(t), as in the following Equation (7):

(7) $$\;\log\left(k\right)+n\;\log\left(t\right)=\log(k_{0}\left(T\right))-{\;m}_{0}\log\left(\varphi \right)\;$$

After rearrangement, it became Equation (8):

(8)  log(φ)=logF(T) − blog(t) 

The **b** is the ratio of the Avrami exponent (***n***) to the Ozawa exponent (m):

b = n/m

$$F(T)\;={\left[{\;k}_{O}\left(T\right)/k\right]}^{\frac{1}{m}}\;$$

**Figure 10:**
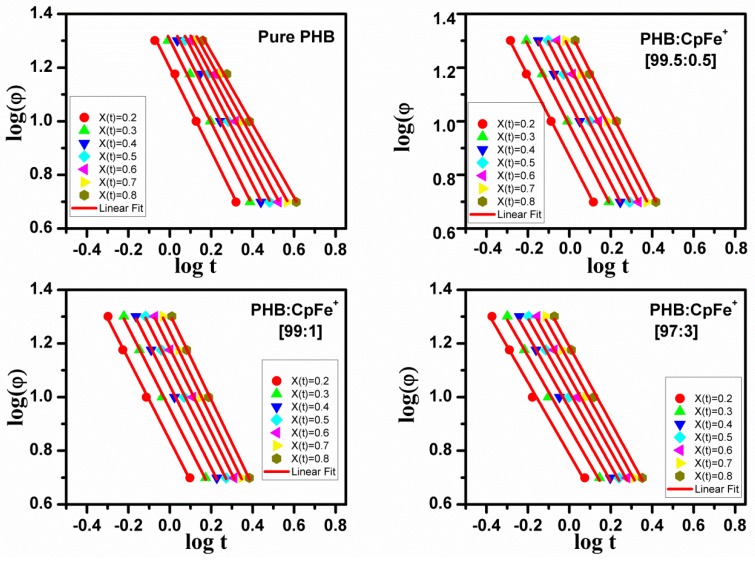
Plots of log(φ) versus log(t) for the pure PHB and PHB/CpFe^+^ nanocomposites that non-isothermally crystallized at a given X(t).

Figure 10 exhibits log(φ) as a function of log(t) at a certain relative crystallinity (20%, 40%, 60%, and 80%) for the PHB/ CpFe^+^ nanocomposites. A good linearity was achieved for all the PHB samples, as shown in Figure 10. The values of logF(T) were determined by the intercept of the lines according to Equation (8) and are reported in Table 4. The F(T) value significantly shifted to higher values with the increase of the degree of the crystallinity. Moreover, for a given X(t), the F(T) values of the PHB/CpFe^+^ nanocomposites decreased with increase of the CpFe^+^ content, indicating a pronounced effect of CpFe^+^.

### 2.8. An Effective Activation Energy of Non-Isothermal Crystallization

Several models in the literature have been proposed to determine the effective activation energy [65]. The isoconversional model is the most applicable model to evaluate the activation energy under a variety of heating and/or cooling rates [66]. The main assumption of this model is that at a constant relative crystallinity, the reaction rate is only a function of the temperature, as follows in Equation (9):

(9) $$\;{\left[\frac{d\;\mathrm{the}\;\ln(X\left(t\right)/t)}{{\mathrm{dT}}^{-1}}\right]}_{x\left(t\right)}=\;-\frac{E_{X\left(t\right)}}{R}\;$$

**Figure 11:**
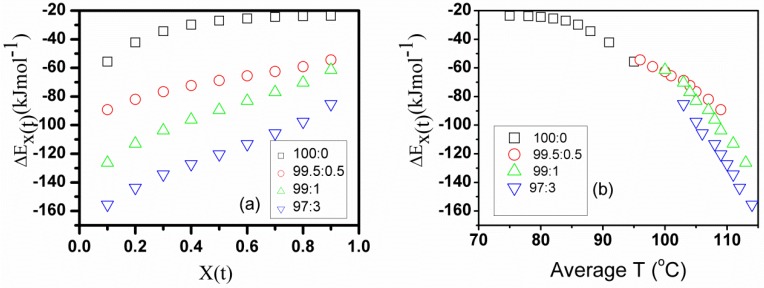
Dependence of the effective activation energy of the pure PHB and PHB/CpFe^+^ nanocomposites on (**a**) X(t) and (**b**) average temperature.

The subscript  X(t) refers to the values corresponding to a given relative crystallinity. Plotting dln(X(t)/t) versus ${\mathrm{dT}}^{-1}$ at different cooling rates at a given X(t) gives a straight line with regression around 0.97. The dependence of the effective activation energy on the relative crystallinity is shown in Figure 11a. It can be discerned that the effective activation energy has a negative value and increases (shifts to a more positive value) with the increase of the relative degree of the crystallinity for the pure PHB and its nanocomposites, Figure 11. This trend was reported previously in the literature.

Vyazovkin [65] stated that the experimental activation energy takes greater negative values at low extents of conversion that correspond to temperatures closer to the melting point. Negative values of the effective activation energy mean that the crystallization rate decreases with increasing temperatures (the anti-Arrhenius process) [67]. Our results showed that the effective activation energy of the pure PHB increased slightly with the extent of the crystallization from −38 to −41 ${\mathrm{kJ}\;\mathrm{mol}}^{-1}$. A similar trend was observed for pure PHB in [68].

In general, the effective activation energy of the pure PHB in its nanocomposites is much lower than that of the pure form. The lowest activation energy value was obtained at the PHB/CpFe^+^ ratio of 97:3. The reduction of the effective activation energy values in the nanocomposites has been attributed to the occurrence of the heterogeneous nucleation mechanism with the organometallic moieties, which can be distinguished in the polarized optical microscopy images, Figure 1.

The activation energy is closely related to the transport of the macromolecular chains to the growing surface and nucleation steps [65]. Figure 11b elucidates the dependence of the effective activation energy on the average temperature. The average temperature can be estimated by an average crystallization temperature related to a given relative crystallinity. As noticed from Figure 11b, the effective activation energy exhibited more negative values at higher average temperatures, which is consistent with the previous report [65]. The combination of the CpFe^+^ moieties with the PHB prompted the formation of more nuclei at high crystallization temperatures, which, consequently, has the lowest effective activation energy.

### 2.9. Thermal Gravimetric Analysis

**Figure 12:**
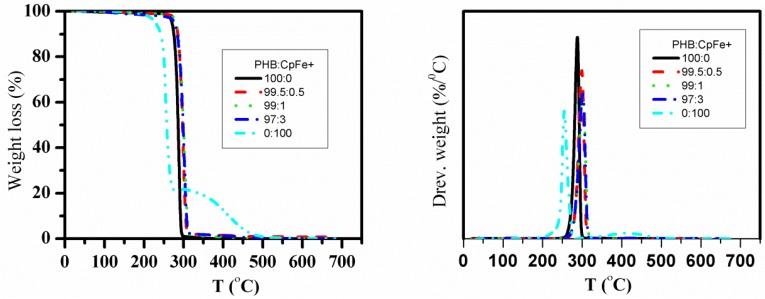
Thermogravimetry and differential thermogravimetry curves of the pure PHB and PHB/CpFe^+^ nanocomposites.

The thermal stability of the PHB in the nanocomposites was determined using TGA and DTGA, as shown in Figure 12, and summarized in Table 5. The maximum degradation temperature of the pure PHB was achieved at 288 °C. Moreover, the CpFe^+^ complex was degraded by two steps. The initial degradation step corresponded to the cleavage of the arene ring, while the second step was comprised of the decomposition of the organic moieties. The maxima degradation temperatures of the CpFe^+^ for the first and second steps were 243 °C and 410 °C, respectively. On the other hand, the PHB/CpFe^+^ nanocomposites decomposed through a one-step degradation mechanism, which was found to be higher than that of the pure PHB by 10 °C. Furthermore, the thermal degradation temperatures of the PHB/ CpFe^+^ nanocomposites were almost constant and were independent of their ratios, as shown in Table 5 This result implies that the introduction of the CpFe^+^ complex improved the thermal stability of the PHB due to the strong chemical interaction, as described previously in the text.

## 3. Materials and Methods

The pure bacterial Poly(3-hydroxybutyrate) (PHB), in the form of white powder, was kindly provided by Dr. Haenggi, Biomer Company, Germany. The PHB was more than 98%, the content of the polyhydroxyvalerate (PHV) was less than 1%, and the remaining cell membrane and membrane lipids were ca. 1%. The weight average molecular weight was about 600,000 g/mol. The melt flow rate (MFR) at 180 °C with 2.16 kg was 10 g/10 min; methylene chloride (99.9%) was supplied from Sigma Aldrich, Germany. No further purification was performed on the materials. The η^6^-arene-η^5^-cyclopentadienyliron complex (CpFe^+^) was prepared in accordance with the previously reported methodology [69]. The structural identity of the iron complex was confirmed using spectroscopic analysis. For example, the ^1^H NMR spectrum exhibited two singlet resonances at 5.6 and 6.9 ppm, which corresponded to the equivalent cyclopentadienyl protons and the complexed aromatic arene protons, respectively. In the meantime, the FTIR spectrum displayed an out of plan bending peak, corresponding to the complexed arene C–H at 816 cm^−1^, while their stretching bands were observed at 3097, 469, and 553 cm^−1^. Furthermore, the frequencies of the complexed cyclopentadienyl C–H stretching band resonated in the range of 2901–2987 cm^−1^.

### 3.1. Preparation of Blends

PHB/CpFe^+^ composites with different weight ratios were prepared through a casting film technique using methylene chloride as a solvent. For each film, approximately 2.00 g of the total sample were mixed and dissolved in 50 mL of methylene chloride with stirring at 40 °C for 45 min to ensure the complete dissolution of both components. After that, the solution was kept at room temperature for 120 min and then casted onto a glass Petri dish at room temperature to slowly evaporate the solvent and to form homogenously a film. The cast film samples were molten between hot melt press at 200 °C for 2 min. The thickness of the specimen was measured using a digital micrometer and was found to be 0.67 ± 0.01 mm.

### 3.2. Characterization

The spherulitic morphologies of the samples were examined on an Olympus CX31 polarizing optical microscope (POM) equipped with a digital camera system, E330. Samples weighing 3–5 mg were melted on glass slips with cover slips to form 20–50 mm thick films. Each specimen was melted at 200 °C for 2 min on a hot stage and then was cooled to the selected crystallization temperature of 100 °C. The polarized optical micrographs were recorded after 1 h annealing at the crystallization temperature of 100 °C.

Wide angle X-ray diffraction, XRD, measurements were conducted by a Shimadzu XRD-6100 X-ray diffractometer with Cu-Kα radiation (λ = 0.154 nm, 40 kV, and 30 mA). The X-ray diffraction patterns were carried out at ambient temperature in the 2θ range of 10–80° with a scanning step and rate of 0.02° and 2°/min, respectively.

The transition temperatures of the samples were detected using a differential scanning colorimeter DSC-Q2000 from TA instruments Co. with a Universal Analysis 2000. The DSC temperature and heat flow were calibrated with indium according to the methodology mentioned previously [70]. All the experiments were carried out under a nitrogen atmosphere (30 mL/min). The samples were first heated from −50 °C to 200 °C to eliminate their thermal history and enhance their thermal contact at a heating rate of 20 °C; subsequently, they were cooled to −50 °C at a rate of 20 °C/min and then were reheated to 200 °C at a rate of 20 °C/min (second heating rate). The data of the first heating run are not discussed here. The crystallization temperature ($T_{C}$) and its enthalpy ($\Delta H_{C}$) were detected through the cooling curve. The glass transition temperature ($T_{g}$) was estimated as the midpoint of the specific heat capacity step from the second heating run. The cold crystallization temperature ($T_{CC}$), the melting temperature ($T_{m}$), and their enthalpies ($\Delta H_{CC}$ and $\Delta H_{m}$) were determined from the respective exothermal and endothermal processes in the DSC second heating run. The overall crystallinity ($\chi_{c}$) of the PHB in the blend was calculated with Equation (1). 

$\;\Delta H_{m}\;$ and  ΔHcc are the experimental melting and cold crystallization enthalpy, respectively. w is the weight fraction of the PHB in the blend, and $\Delta H_{m}^{{}^{\circ}}$ is the melting enthalpy of 100% crystalline of PHB, which is taken as 146 J/g [15].

For the study of the non-isothermal crystallization, the samples were heated to 200 °C rapidly and held for 2 min, and then, the DSC curves were recorded during cooling at various cooling rates (5, 10, 15, and 20 °C/min). The crystallization temperature during cooling ($T_{C}$) and the enthalpy of crystallization on cooling ($\Delta H_{C}$) were determined from these scans. 

Thermogravimetric analysis, TGA, measurements were conducted using a TA Instruments SDT-Q600 thermal analyzer at a heating rate of 20 °C/min under a nitrogen atmosphere with a purge of 20 mL/min from 20 to 700 °C. The sample mass was about 2–5 mg, and it was placed in an alumina crucible.

## 4. Conclusions

This study represents the first example of employing the cationic cyclopentadienyliron (CpFe^+^) complex as a nucleating agent for the enhancement of the properties of the PHB biodegradable polymer. The influence of this metallic complex on the crystallization behavior of the bacterial PHB has been studied profoundly. The new nucleating agent displayed an excellent distribution into the polymer matrix without any sign of agglomeration. Meanwhile, the blending process was assisted by the coordination of the CpFe^+^ with the ester carbonyl of the polymer chains. Furthermore, the incorporation of the CpFe^+^ had a multitude of outcomes. For instance, merging the cationic organoiron complex into the PHB materials shifted the melt crystallization temperature 26 °C higher than that of its pure analogue. It also promptly increased the crystallization rate of the bacterial PHB by 87% with no influence on the polymeric crystal lattice. The isothermal and non-isothermal crystallization behaviors of the polymer have been elucidated using the Avrami model, the modified Avrami model, and the combination Avrami and Ozawa model. In addition, the presence of the cationic nucleating agent lowered the effective activation energy of the PHB, owing to its heterogeneous nucleation mechanism with the polymer matrix, which is an indication of a faster crystallization rate that leads to a shortened production cycle time for the polymer processing techniques. All these results signify that the CpFe^+^ is a superior novel nucleating agent and that the complex has been deemed to be a good candidate for high-temperature polymer processing techniques and commodity applications.

## Figures and Tables

**Table 1:** Thermal parameters of the pure PHB and PHB/CpFe^+^ nanocomposites from DSC (cool and second heating) curves.

Name of PHB Samples		$T_{c}\;\pm \;1$	Width	$\Delta H_{c\;}\;\pm \;5$	$T_{g}\;\pm \;2$	$\;\Delta C_{p}$	$T_{cc}\;\pm \;1$	$\Delta H_{cc}\;\pm \;5$	$T_{m}\;\pm \;1$	width	$(\Delta H_{m}$ $-\Delta H_{cc})\;\pm \;5$	$\;\chi_{c}$
		°C	°C	J/g	°C	J/g°C	°C	J/g	°C	°C	J/g	%
**PHB:CpFe+**	**100:0**	71		59	3	0.21	95	8	170		72	50
	**99.5:0.5**	97	13	72	2	0.16	105	4	170	7	87	60
	**99:1**	97	13	72	2	0.17	104	7	169	7	86	60
	**97:3**	97	12	73	2	0.16	105	4	169	6	87	61

**Table 2:** Isothermal crystallization kinetics parameters of the pure PHB and PHB/ CpFe^+^ nanocomposites based on the Avrami model.

Name of PHB Samples	$\;T_{c}$	n	logk	$\;k\times 10^{-2}$	$\;R^{2}$	$\;t_{0.5}$	$\;t_{0.99}$
	°C			min^−1^		Min	Min
**PHB:CpFe+** **(100:0)**	90	3	−0.23	59. 19	1	1.07	2.1
	94	2	−0.24	58.2	1	1.08	2.52
	100	2	−0.62	24.01	1	1.71	4.05
	118	2	−2.45	0. 35	1	11.5	29
	120	2	−2.80	0.16	1	16.7	37.2
	122	2	−2.80	0.16	1	18.6	37.7
	124	2	−3.13	0.055	1	19.2	39.3
**99.5:0.5**	120	2	−1.41	3.88	1	4.05	9.63
	124	2	−2.29	0.52	1	9.75	21.4
	128	2	−2.98	0.1	1	18.1	38.3
**99:1**	120	2	−0.99	10.06	1	2.69	6.58
	124	2	−1.63	2.33	1	5.43	13.3
	128	2	−2.53	0.29	1	12.7	29
**97:3**	120	2	−0.85	14.23	1	2.31	5.53
	124	2	−1.62	2.37	1	5.4	13.2
	128	2	−2.68	0.21	1	13	28.2

**Table 3:** Non-isothermal crystallization kinetics parameters of the pure PHB and PHB/CpFe^+^ nanocomposites based on the modified Avrami model.

Name of PHB Samples		φ	$\;\Delta t_{inc}$	$T_{c}\;$	$\Delta H_{c}\;$	n	$\;\log k_{t}$	$\;k_{t}\times 10^{-2}$	$\;K_{c}$	$\;R^{2}$	$\;t_{0.5}$	$\mathrm{CRP}\times 10^{-2}$
		°C /min	min	°C	J/g			min^−1^	min^−1^		Min	
**PHB:CpFe+**	**100:0**	5	16.9	102	83.4	3	−1.3	5.13	0.55	1	2.5	2.72
		10	9.14	91	72.1	3	−0.86	13.83	0.81	1	1.8	
		15	6.29	83	67.5	3	−0.59	25.63	0.91	1	1.5	
		20	4.83	76	65.3	3	−0.37	42.76	0.95	1	1.2	
	**99.5:0.5**	5	15.7	112	91.5	3	−0.99	10.06	0.63	1	2	6.36
		10	8.09	109	87.9	3	−0.24	58.16	0.91	1	1.1	
		15	5.53	105	85.4	3	0.11	131.2	0.99	1	0.8	
		20	4.24	102	83.3	3	0.35	224.4	1.01	1	0.7	
	**99:1**	5	15.6	112	93.8	3	−0.97	10.71	0.64	1	1.9	5.12
		10	8.07	107	87.4	3	−0.35	44.5	0.92	1	1.2	
		15	5.51	104	85.4	3	−0.04	90.49	0.99	1	0.9	
		20	4.21	101	83.6	3	0.16	145.8	1.02	1	0.8	
	**97:3**	5	15.8	113	90.9	3	−0.90	12.49	0.66	1	1.8	6.5
		10	8.06	110	85.8	3	−0.15	70.08	0.97	1	1	
		15	5.5	106	84	3	0.17	148.3	1.02	1	0.8	
		20	4.21	104	81.9	3	0.4	251.7	1.05	1	0.6	

**Table 4:** F(T) and b values by MO approach for pure PHB and PHB/CpFe^+^ nanocomposites.

Name of PHB Samples		Kinetics Parameter	X(t)			
			0.2	0.4	0.6	0.8
**PHB:CpFe+**	**100:0**	F(T)	15.83	23.59	29.10	33.80
		b	−1.56	−1.52	−1.45	−1.36
		$\;R^{2}$	0.997	0.993	0.993	0.995
	**99.5:0.5**	F(T)	7.239	8.903	10.047	11.122
		b	−1.496	−1.509	−1.522	−1.522
		$\;R^{2}$	0.999	0.999	0.999	0.998
	**99:1**	F(T)	6.978	8.669	9.825	10.889
		b	−1.512	−1.535	−1.565	−1.602
		$\;R^{2}$	0.998	0.999	0.999	0.999
	**97:3**	F(T)	6.546	7.965	8.940	9.882
		b	−1.339	−1.362	−1.384	−1.414
		$\;R^{2}$	0.993	0.994	0.995	0.995

**Table 5:** Thermal stability parameters of PHB and PHB/ CpFe^+^ nanocomposites.

Name of PHB Samples		PHB Phase		
		$\;T_{onset}\;$	$\;T_{max}$	$\;T_{end}$
**PHB:CpFe+**	**100:0**	280	288	293
	**99.5:0.5**	287	297	303
	**99:1**	287	297	303
	**97:3**	286	298	305
	**90:10**	287	300	306
	**0:100**	200 354	243 410	255 455
